# The role of leptin in the control of insulin-glucose axis

**DOI:** 10.3389/fnins.2013.00051

**Published:** 2013-04-08

**Authors:** Marie Amitani, Akihiro Asakawa, Haruka Amitani, Akio Inui

**Affiliations:** Department of Psychosomatic Internal Medicine, Kagoshima University Graduate School of Medical and Dental SciencesKagoshima, Japan

**Keywords:** food intake, leptin, adipo-insulin axis, leptin gene therapy

## Abstract

Obesity and diabetes mellitus are great public health concerns throughout the world because of their increasing incidence and prevalence. Leptin, the adipocyte hormone, is well known for its role in the regulation of food intake and energy expenditure. In addition to the regulation of appetite and satiety that recently has attracted much attentions, insight has also been gained into the critical role of leptin in the control of the insulin-glucose axis, peripheral glucose and insulin responsiveness. Since the discovery of leptin, leptin has been taken for its therapeutic potential to obesity and diabetes. Recently, the therapeutic effects of central leptin gene therapy have been reported in insulin-deficient diabetes in obesity animal models such as ob/ob mise, diet-induced obese mice, and insulin-deficient type 1 diabetes mice, and also in patients with inactivating mutations in the leptin gene. Herein, we review the role of leptin in regulating feeding behavior and glucose metabolism and also the therapeutic potential of leptin in obesity and diabetes mellitus.

## Introduction

Obesity and diabetes mellitus are important public health concerns throughout the world because of their increasing incidence and prevalence. The pooled prevalence of obesity is currently as high as 23.5%, and the general prevalence of diabetes among adults in the US is 6.3% (Sullivan et al., 2005). Obese individuals have an increased risk of morbidity because of the various related disorders, including diabetes, cardiovascular events, stroke, cancer, and obstructive sleep apnea (Must et al., 1999; Field et al., 2001; Polesel et al., 2009; Shah and Roux, 2009).

Food intake is controlled by a complex regulatory network that depends on the central regulation of energy homeostasis. The signals to regulate food intake are ultimately integrated or coordinated by central mechanisms, particularly in the hypothalamus. There are many factors to consider in the hypothalamic regulation of food intake, and the interactions between adiposity and the central neuropeptidergic cascade downstream of leptin are increasingly being studied.

Since the discovery of leptin, it has been expected the therapeutic potential for obesity and diabetes. Leptin is known as a key appetite-regulating hormone, which effects on appetite, energy expenditure, behavior, and glucose metabolism. Much evidence suggests that insulin and leptin act in the brain as adiposity negative feedback signals (Morton and Schwartz, 2011).

Indeed, recent studies revealed that leptin has the effect to normalize hyperglycemia and hyperinsulinemia and to increase insulin sensitivity. (Kamohara et al., 1997).

The central leptin gene therapy has also been effective to insulin-deficient diabetes in obesity animal models such as ob/ob mise, diet-induced obese mice, and insulin-deficient type 1 diabetes mice (Kojima et al., 2009), and in patients with inactivating mutations in the leptin gene (Farooqi et al., 1999). Thus, leptin gene therapy is expected to be an effective therapeutic option for obesity, and diabetes.

Herein, we review the role of leptin in regulating feeding behavior and glucose metabolism and also the therapeutic potential of leptin in obesity and diabetes mellitus.

## Leptin

Leptin is a 16-kDa protein hormone, which is secreted by adipocytes. Plasma leptin concentration increases in proportion to body fat mass, and regulate food intake and energy expenditure to maintain body fat stores. (Zhang et al., 1994; Campfield et al., 1995; Halaas et al., 1995; Pelleymounter et al., 1995; Kamohara et al., 1997; Elias et al., 1999). Circulating leptin is secreted into the blood-stream and reaches to the brain through the brain-blood barrier (BBB) and cebrospinal fluid (CSF) barrier. Leptin act in the hypothalamus, where leptin inhibits neuropeptide Y (NPY) neurons and causes anorexia (Elmquist et al., 1999). These will be discussed in the following sections.

## Central regulation of food intake and energy expenditure

The regulation of food intake is coordinated by central mechanisms, particularly in the hypothalamus. The hypothalamus plays an important role in regulating appetite and energy expenditure. Particularly, the arcuate nucleus of the hypothalamus arcuate nucleus (ARC) is critical for appetite regulation and the mechanism of obesity. There are many factors to consider in the hypothalamic regulation of food intake, e.g., melanin-concentrating hormone (MCH), NPY, agouti-related protein (AgRP), proopiomelanocortin (POMC), cocaine-and-amphetamine responsive transcript (CART), oxytocin, arginine vasopressin, brain-derived neurotrophic factor (BDNF), serotonin (5-HT), corticotropin-releasing factor (CRF) and the urocortins (UCN1, UCN2, UCN3).

The ARC projects to other hypothalamic sites, such as the periventricular nucleus (PVN), dorsomedial (DMH) hypothalamic nuclei, and the lateral hypothalamic area (LHA). The neurons include POMC, NPY, and AgRP are located in ARC. The other neurons include α-melanocyte–stimulating hormone (αMSH), which are the melanocortin peptides that are derived from POMC. NPY/AgRP neurons release the orexigenic peptides NPY and AgRP, lead to increased food intake (Williams et al., 2011; Xu et al., 2011). By contrast, POMC neurons synthesize and secrete an anorexigenic peptide, α-MSH, which activates melanocortin receptors and leads to decreased food intake. Central or peripheral administration of leptin increases POMC/CART mRNA expression, and NPY and AgRP mRNAs are inhibited by leptin administration.

The other important factors are CRF and 5-HT systems. CRF and endogenous CRF receptor ligand urocortin are feeding-inhibitory peptides localized at the PVN. Urocortin binds both CRF type 1 and 2 receptors but shows a higher affinity for CRF type 2 receptor than type 1 receptor. CRF type 2 receptors are related to the stress-induced alterations of gastrointestinal functions (Asakawa et al., 1999; Ushikai et al., 2011; Fujimiya et al., 2012).

Central 5-HT systems also play an important role in the suppression of feeding. Particularly, the 5-HT2C receptor (5-HT2CR) and 5-HT1B receptors (5-HT1BRs) are widely expressed in the central nervous system (CNS), mediating the anorexigenic activity of endogenous melanocortin receptor agonists and antagonists at the melanocortin 4 receptor (MC4R) (Lam et al., 2008; Marston et al., 2011). 5-HT acts on 5-HT2CR in POMC neurons to regulate feeding and insulin sensitivity via MC4R signaling pathways (Zhou et al., 2007). 5-HT depletion does not impair the anorectic effects of leptin, which suggests that leptin does not directly cause CNS serotonin neurons to influence appetite (Lam et al., 2011).

In the peripheral feed-regulating peptide, ghrelin act conversely with leptin. Ghrelin is an endogenous agonist at the growth hormone (GH) secretagogue receptor and is a member of the motilin-related family of regulatory peptides. In addition to its ability to stimulate GH secretion and gastric motility, ghrelin stimulates appetite and induces a positive energy balance that leads to weight gain. Leptin and ghrelin are complementary, yet antagonistic, signals that reflect acute and chronic changes in the energy balance. Endocrine and vagal afferent pathways are involved in these actions of ghrelin and leptin (Inui et al., 2004). Ghrelin activates NPY/AgRP neurons in the PVN and suppresses POMC neuronal activity to regulate food intake (Cowley et al., 2003). As to glucoregulatory functions, increased leptin levels are associated with obesity and type 2 diabetes, while decreased ghrelin levels are associated with obesity and insulin resistance rather than type 2 diabetes. Glucose infusion rate positively correlated with plasma ghrelin and negatively correlated with serum leptin levels (Zhang et al., 2012).

## Leptin transgenic animal model

The central or peripheral administration of leptin to rodents reduces food intake and increases energy expenditure (Ahima et al., 2000). In transgenic animal models, such as leptin-deficient (ob/ob) mice and LepRb-deficient (db/db) mice, exhibit the development of marked obesity, insulin resistance, hyperinsulinemia, impaired glucose homeostasis, and diabetes (Schwartz et al., 1996; Cohen et al., 2001; McMinn et al., 2005). By contrast, transgenic and brain-specific reconstitution of LepRb in db/db mice improves obesity and daibetes. Fasting rodents with reduced leptin levels and ob/ob mice exhibit decreased level of hypothalamic POMC mRNA, which is normalized by exogenous leptin administration that subsequently improves obesity and diabetes.

## Leptin and synaptic plasticity

Recent findings suggest that leptin exerts plastic effects on neural connections and that leptin might function as a developmental cue for brain development (Ahima et al., 1998; Bouret et al., 2004). Leptin is required for normal development of ARC pathways, and this developmental activity of leptin is restricted to a neonatal window of maximum sensitivity, which corresponds to a period of elevated leptin secretion (Bouret and Simerly, 2004). Leptin levels during the perinatal period are crucial for the development of metabolic systems that are involved in energy homeostasis, food seeking and reward behaviors. Rodents exhibit a postnatal leptin surge, in whitch circulating leptin levels increase around postnatal day 5 and peak between postnatal day 9 and 10 (Granado et al., 2011, 2012). In animal studies, the treatment of rodents with exogenous leptin does not affect milk intake or metabolic rates until after weaning (Mistry et al., 1999; Proulx et al., 2002). Blocking the postnatal leptin surge results in long-term leptin insensitivity and increased susceptibility to diet-induced obesity during adulthood (Attig et al., 2008). The offspring of obese rats display an amplified and prolonged neonatal leptin surge, which is accompanied by elevated leptin mRNA expression in their abdominal white adipose tissue (Kirk et al., 2009). Maternal undernutrition markedly reduces the postnatal surge of plasma leptin, which particularly disturb the hypothalamic wiring and the gene expression of the anorexigenic POMC neurons (Delahaye et al., 2008). In the absence of leptin, the AgRP/NPY and α-MSH pathways are severely disrupted in adult ob/ob mice (Bouret and Simerly, 2004). By contrast, when leptin is delivered systemically to ob/ob mice, the synaptic density rapidly normalizes; effect is detectable within 6 hour, which is several hours before leptin's effect on food intake (Pinto et al., 2004). In the obesity and hyperleptinemic rat, phenotype impaird hippocampal synaptic plasticity (Grillo et al., 2011a,b).

## Leptin signaling

### Leptin isoforms

Leptin exert its antiobesity effect via leptin receptor (LepR) in (ARH) (Fei et al., 1997; Bouret and Simerly, 2004). There are six isoforms of LepRs (OB-R): OB-Ra, OB-Rb, OB-Rc, OB-Rd, OB-Re, and OB-Rf (Fruhbeck, 2006). The short form of LepR is widely expressed in multiple tissues (Elmquist et al., 1998). The longer form of the LepR encodes a protein with a longer cytoplasmic domain (OB-Rb). OB-Rb is a member of the class 1 cytokine receptor family and considered the main isoform involved in the transduction of intra cellular signals (Mantzoros et al., 2011; Marroqui et al., 2012). OB-Rb is expressed highly in PMV, ARH, DMH, VMH, MEPO (medial preoptic nucleus) (Gautron and Elmquist, 2011).

### JAK/STAT pathway

**Figure 1:**
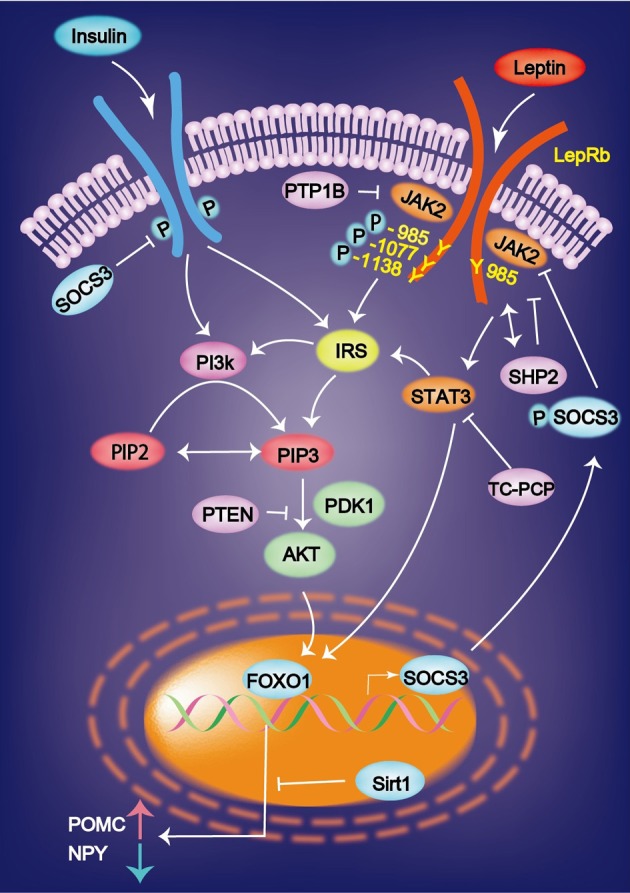
**Leptin binds to the leptin receptor (LepRb) and activates the receptor-associated kinase JAK2 via transphosphorylation and phosphorylates three tyrosine residues (Y985, Y1077, and Y1138).** Leptin-induced mRNA expression of JAK-STAT is inhibited by SOCS3. Insulin and leptin regulate the expression of AgRP and POMC via Foxo1 and signal transducer and activator of transcription factor Stat3. Sirt1 suppresses the Foxo1-dependent expression of the orexigenic neuropeptide AgRP. AgRP, agouti-related protein; FOXO1, Forkhead box O1; IRS, insulin receptor substrate; PI3K, phosphatidylinositol 3 kinase; PIP3, phosphatidylinositol 3, 4, 5-triphosphate.

Leptin signaling via the long form of OB-Rb is intracellularly coupled with the janus kinase (JAK)–signal transducer and activator of transcription (STAT) pathway (Jak/STAT pathway). Leptin binds to OB-Rb and activates the receptor-associated kinase JAK2 via transphosphorylation and phosphorylates three tyrosine residues (Y985, Y1077, and Y1138 in mice). The signals emanating from the LepR Tyr985 control hepatic insulin sensitivity. Lack of LepRb-Tyr985 signaling enhances whole-body insulin sensitivity partly through increased suppression by insulin of hepatic glucose production. Leptin stimulates JAK2-dependent phosphorylation and nuclear translocation of the transcription factor signal transducer and activator of STAT3 and STAT5 the phosphorylated Y1138 on LepRb. This JAK2/STAT3 pathway is required for leptin control of energy balance and body weight. pSTAT3 translocates to the nucleus, where it increase the expression of POMC and inhibits that of NPY (St-Pierre and Tremblay, 2012) (Figure 1). Defects in leptin signaling lead to leptin resistance, which is a primary risk factor for obesity.

STAT3 regulates glucose homeostasis by suppressing the expression of gluconeogenic genes in the liver. Plasma insulin stimulates tyrosine phosphorylation of STAT3 in the liver, and hepatic IL-6 that is induced by the brain-insulin action is necessary for the activation of STAT3 (Inoue et al., 2006). The suppressed hepatic glucose production and expression of gluconeogenic genes induced by intracerebral ventricular insulin infusion is impaired in liver-specific STAT3 deficiency mice and in IL-6 deficient mice (Inoue et al., 2006). These results demonstrate that IL-6-STAT3 signaling in the liver contributes to insulin action in the brain, which suppresses hepatic glucose production.

### PI3K

Another important pathway is PI3K. JAK2 stimulates the phosphorylation of insulin receptor substrate (IRS) 1 or 2. IRS then activates PI3K, leading to the production of phosphatidylinositol 3, 4, 5-triphosphate (PIP3). PIP3 formation activates Akt, which subsequently prevents Forkhead box O (FoxO)-1 from inhibiting the expression of POMC and from stimulating the expression of NPY and AgRP. Overall, PI3K promotes the effects of leptin by inhibiting FoxO1 (Wauman and Tavernier, 2011; St-Pierre and Tremblay, 2012). A mutation in the gene encoding the protein kinase AKT2/PKB-β reveals the autosomal dominant inheritance of severe insulin resistance and diabetes mellitus (George et al., 2004). The level of Sirt1 protein is increased in the hypothalamus during feeding, which decreases the FoxO1-dependent expression of AgRP and results in the cessation of feeding (Sasaki et al., 2010).

### SOCS3

Recently, a new family of molecules, called suppressors of cytokine signaling (SOCS), has been identified. Leptin-induced mRNA expression of JAK-STAT is inhibited by SOCS3 in insulin (INS)-1 β-cells and human pancreatic islets *in vitro* and in the pancreatic islets of ob/ob mice *in vivo* (Laubner et al., 2005). SOCS3 inhibits basal and STAT3/5b-dependent rat preproinsulin 1 gene promoter activity in INS-1 cells (Laubner et al., 2005). The SOCS molecules might play an important role in the development of leptin resistance that is observed in the CNS and in the endocrine pancreatic beta-cells during obesity (Seufert, 2004). These results indicate that SOCS3 represents a transcriptional inhibitor of preproinsulin gene expression, which is induced by leptin via JAK-STAT3/5b signaling in pancreatic β-cells (Laubner et al., 2005).

### Phosphatases

There are five main phosphatases involved in leptin signaling: SHP2, PTEN, PTP1B, and the recently implicated TCPTP and RPTP epsilon. With the exception of SHP2 that globally promotes leptin signaling by coupling to ERK kinase, the other four phosphatases work by inhibiting leptin signaling (St-Pierre and Tremblay, 2012).

Phosphatase and tensin homolog deleted on chromosome 10 (PTEN) is a class 1 Dsp known mainly for its tumor suppressor functions (Julien et al., 2011). PTEN plays an important role in leptin signaling because it is a negative regulator of the PI3K pathway (St-Pierre and Tremblay, 2012). By enhancing insulin-PI3K signaling via deletion of PTEN, β-cell mass and function lead to increase. PTEN expression in islets was upregulated in both models of type 2 diabetes. PTEN exerts a critical negative effect on both β-cell mass and function (Wang et al., 2010a).

## Leptin resistance

Although current pharmacological and behavioral treatments for obesity cause initial weight loss, this effect is transient and generally followed by weight regain, which is associated with leptin resistance (Yanovski and Yanovski, 2002). Despite an increase in plasma leptin levels, rats that are fed a high-fat diet become obese (Akiyama et al., 1996).

The mechanism of leptin resistance has been revealed. The first cause of leptin resistance is impaired leptin transport across the BBB. The second cause of leptin resistance is impaired leptin signal transduction in neurons, including SOCS3, PTP1B. As the other mechanism, the hypothalamic inflammation, the role of endoplasmic reticulum stress and defective autophagy are suggested to be involved.

Leptin transport across the BBB is impaired in obesity (Burguera et al., 2000). The permeability of the BBB to leptin is decreased in high-fat diet-induced obese rats despite increased plasma leptin levels (Burguera et al., 2000). OB-Ra and OB-Rc are highly expressed in the choroid plexus and microvessels, where these receptors are assumed to play a role in leptin uptake or efflux from the cerebrospinal fluid (CSF) and in receptor-mediated transport of leptin across the BBB (Hileman et al., 2002); by contrast another study indicates that leptin transport across the BBB is not mediated by a product of the LepR gene (Banks et al., 2002).

**Figure 2:**
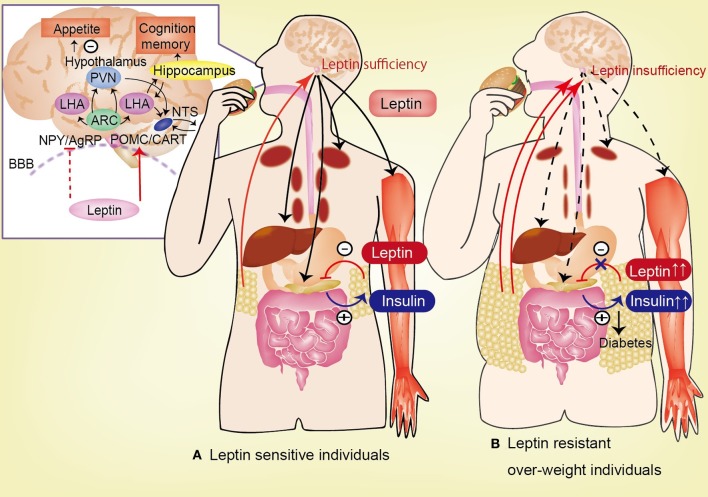
**Circulating leptin is correlated with the degree of adiposity and is transported across the blood-brain barrier (BBB).** In hypothalamus, leptin activates POMC and CART neurons, and inhibit NPY and AgRP neurons, lead to anorexia. Leptin has effect to feeding behavior, appetite, insulin-glucose axis, and cognitive function. **(A)** In leptin sensitive individuals, leptin inhibits insulin biosynthesis and secretion from pancreatic β-cells. By contrast, insulin stimulates leptin secretion from adipose tissue. Leptin stimulates hepatic gluconeogenesis and hepatic insulin sensitivity via the hepatic branch of the vagus nerve. Additionally, leptin increases glucose uptake in the skeletal muscle, heart, and brown adipose tissue (BAT) via the sympathetic nervous system. **(B)** In leptin resistant over-weight individuals, the permeability of the BBB to leptin is decreased in high-fat diet-induced obesity despite the increase in plasma leptin levels. This impaired transport of leptin across the BBB is one of the causes of leptin resistance. Insufficiency of leptin signaling in the hypothalamus (induced by hyperleptinemia in obese subjects), causes hyperglycemia and hyperinsulinemia, which lead to diabetes mellitus. AgRP, agouti-related protein; ARC, arcuate nucleus; CART, cocaine-and-amphetamine responsive transcript; LHA, lateral hypothalamic area; NPY, neuropeptide Y; POMC, proopiomelanocortin; BBB, blood-brain barrier.

Hyperleptinemia, which either manifests gradually in association with age-related obesity or is produced rapidly by the consumption of energy-enriched diets, is associated with decreased brain leptin concentrations in rodents and humans (Banks et al., 1999; Burguera et al., 2000; Banks and Farrell, 2003; Banks et al., 2006; Suzuki et al., 2008; Ziylan et al., 2009; Kalra, 2011). Increased blood levels of leptin fail to reduce feeding or to be reflected in increased levels of leptin in the CSF. If leptin is delivered directly into the brain, it reduces feeding, but delivery into the blood is essentially without effect (Kastin and Pan, 2006). Insufficient leptin signaling in the hypothalamus, which is caused by either decreased availability of leptin for transport to the hypothalamus (in the case of leptinopenia), or restricted leptin entry across the BBB (imposed by hyperleptinemia in obese subjects), is primarily responsible for inducing hyperglycemia and hyperinsulinemia; the persistence of these pathophysiologic sequelae culminates in diabetes mellitus (Kalra, 2011; Figure 2).

## Leptin and glucose metabolism

Leptin plays an important role to regulate energy balance and also affects peripheral glucose homeostasis and locomotor activity. There are notable reports to discuss the leptin and glucose metabolism. The ARC has been proposed as an important site of leptin action. Unilateral restoration of leptin signaling in the ARC of LepR null allele [Lepr (neo/neo) mice] leads to a modest decrease in body weight and food intake. In contrast, unilateral reactivation markedly improved hyperinsulinemia and normalized blood glucose levels and locomotor activity (Coppari et al., 2005). These data demonstrate that ARC in the brain have the role to mediate the antidiabetec actions of leptin in models of type 2 diabetes.

In the other study, the expression of ObRb only in POMC neurons leads to a marked decrease in energy intake and a modest reduction in body weight (Huo et al., 2009). The blood glucose levels are normalized independent of changes in food intake and body weight. Furthermore, physical activity is greatly increased despite profound obesity. These results suggest that leptin signaling in POMC neurons in the ARC is crucial to stimulate activity and prevent diabetes in db/db mice (Huo et al., 2009).

Leptin and insulin signaling regulate food intake by controlling the expression of orexigenic and anorexigenic neuropeptides in ARH via FoxO1 and the STAT-3. Insulin regulates the activity of the Forkhead transcription factor (Foxa-2) by Akt-mediated phosphorylation (Wolfrum et al., 2003). Foxa2 is a downstream target of insulin-signaling and plays an important role in the integration of metabolic signals, adaptive behavior and physiological responses (Silva et al., 2009). Foxa2 binds to MCH and orexin promoters and stimulates their expression, which leads to increased food consumption, metabolism and insulin sensitivity.

AMP-activated protein kinase (AMPK) is an intracellular energy sensor and regulator of food intake. AMPK regulates food intake by responding to hormonal and nutrient signals in the hypothalamus (Minokoshi et al., 2004). AMPK regulates energy expenditures by modulating NAD^+^ metabolism and sirtuin1 (Sirt1) activity (Canto et al., 2009). AMPK also regulates energy levels by switching off ATP-consuming pathways and switching on ATP-producing pathways, such as glucose uptake and fatty acid oxidation. Leptin inhibits hypothalamic AMPK activity and restricts food intake. There are four mechanisms to be considered: first, ghrelin stimulates AMPK in NPY/AgRP in the arcuate nucleus and in the ventromedial hypothalamic nucleus and activates an AMPK-dependent presynaptic pathway that sustains AgRP neuron firing via a local synaptic memory system. Second, adiponectin stimulates hypothalamic AMPK. Third, hypothalamic AMPK controls energy expenditures via thyroid hormone or leptin (Stark et al., 2013). Leptin treatment significantly suppresses NPY secretion in the cells. Leptin inhibits AMPK activity and activates acetyl-coenzyme acarboxylase in NPY neurons. When AMPK is inhibited by compound C or phosphatidylinositol 3 kinase (PI3K), the leptin-mediated decrease in NPY secretion is prevented (Dhillon et al., 2011).

Defects in glucose sensing also result in obesity. Glucose starvation inhibits the ability of leptin to stimulate tyrosyl phosphorylation and inhibits the activation of JAK2 and STAT3 *in vitro* (Su et al., 2012). Glucose dose-dependently enhances leptin signaling. By contrast, glucose does not enhance GH-stimulated phosphorylation of JAK2 and STAT5 (Su et al., 2012). Glucose starvation or 2-deoxyglucose induced inhibition of glycolysis activates AMPK and inhibits leptin signaling. The inhibition of AMPK restores the ability of leptin to stimulate STAT3 phosphorylation. AMPK inhibits leptin signaling and blocks the ability of glucose to enhance leptin signaling (Su et al., 2012).

Sirt1 improves insulin sensitivity *in vitro*. In an animal model, hypothalamic Sirt1 protein increases food intakes, and this effect is shown in diet-induced obese mice and db/db mice. Forced expression of wild-type Sirt1 in the mediobasal hypothalamus by adenovirus microinjection suppresses FoxO1-induced hyperphagia, which is a model for central insulin resistance (Sasaki et al., 2010). FoxO1 is a transcription factor that controls liver metabolism and pancreatic β-cell function. The expression of the genes encoding AgRP and POMC neuropeptides is regulated by insulin and leptin via Foxo1 and the STAT factor Stat3 (Kitamura et al., 2006). Hypothalamic Sirt1 protein levels increase during feeding, and the forced expression of wild-type Sirt1 in the mediobasal hypothalamus suppresses FoxO1-induced hyperphagia. Sirt1 suppresses AgRP promoter activity, which leads to decreased FoxO1-dependent expression of orexigenic neuropeptide AgRP and the cessation of feeding (Nakae et al., 2006; Sasaki et al., 2010). Resveratrol is a pharmacological activator of Sirt1, which improves the life span and health of mice that are fed a high fat diet (Baur et al., 2006). Resveratrol activates Sirt1 activity and suppress the FoxO1-dependent expression of the orexigenic neuropeptide AgRP (Sasaki et al., 2010).

Leptin treatment improves insulin resistance and hyperglycemia in a specific mouse model of type 2 diabetes MKR (transgenic overexpression of a skeletal muscle dominant-negative IGF-I receptor with a lysine-to-arginine amino acid). Leptin treatment enhanced hepatic insulin sensitivity of the MKR mice by suppression of hepatic glucose production during the hyperinsulinemiceuglycemic clamp. Additionally, leptin reduced gene expression of key regulator of glucose metabolism such as G6Pase, a gluconeogenic enzyme in liver (Toyoshima et al., 2005).

## The effect of leptin on liver and peripheral tissues

In humans and in rodents, various OB-R isoforms are widely distributed in many organs, including the pancreas, liver, heart, kidney, adipose tissue, and brain. Leptin regulates glucose homeostasis and hepatic insulin sensitivity, as well as food intake and energy expenditures by activating the long form of the LepRb. The central administration of leptin increases glucose turn-over and glucose uptake in peripheral tissues, [such as the skeletal muscle, heart, and brown adipose tissue (BAT)], via the sympathetic nervous system.

Leptin stimulates hepatic gluconeogenesis and hepatic insulin sensitivity via the hepatic branch of the vagus nerve. In LepR-deficient Koletsky rats, the adenovirally induced expression of LepRs in ARC nucleus improves peripheral insulin sensitivity via enhanced suppression of hepatic glucose production, with no change in insulin-stimulated glucose uptake or disposal. This effect is associated with increased insulin signal transduction via phosphatidylinositol-3-OH kinase in the liver (but not in the skeletal muscle) and with reduced hepatic expression of the gluconeogenic genes, glucose-6-phosphatase and phosphoenolpyruvate kinase (German et al., 2009). Moreover, the effects of hypothalamic leptin signaling on hepatic insulin sensitivity are blocked by selective hepatic vagotomy, which suggests that the effect of hypothalamic leptin on the liver is dependent on the hepatic branch of the vagus nerve (German et al., 2009).

Leptin, insulin, and nutrient-related signals in the CNS improve hepatic insulin sensitivity however, the mechanism by which CNS leptin signaling normalizes diabetic hyperglycemia and whether this process involves leptin-dependent effects on hepatic glucose production or tissue glucose uptake are unknown (German et al., 2011). Leptin action in the brain potently suppresses hepatic glucose production while increasing tissue glucose uptake despite persistent, severe insulin deficiency (German et al., 2011). The hyperinsulinemia in *ob/ob* mice might be caused by the impairment of leptin signaling in pancreatic β-cells and contributes to obesity and insulin resistance. The polyethylene glycolylated mouse leptin antagonist (PEG-MLA)-treated mice exhibit increased fasting and glucose stimulated plasma insulin levels, decreased whole-body insulin sensitivity, elevated hepatic glucose production, and impaired insulin-mediated suppression of hepatic glucose production (Levi et al., 2011).

The decrease of body fat stores in uncontrolled insulin-deficient diabetes results in marked reduce of plasma leptin levels (German et al., 2010). Physiologic leptin replacement prevents insulin resistance in uncontrolled diabetes via a mechanism unrelated to changes in food intake or body weight. This effect is associated with reduced total body fat and hepatic triglyceride content, preservation of lean mass, and improved insulin signal transduction via the insulin receptor substrate-phosphatidylinositol-3-hydroxy kinase pathway in the liver, but not in skeletal muscle or adipose tissue (German et al., 2010). Although physiologic leptin replacement lowers blood glucose levels only slightly, it fully normalizes elevated plasma glucagon and corticosterone levels and reverses the increased hepatic expression of gluconeogenic enzymes characteristic of rats with uncontrolled diabetes (German et al., 2010). Furthermore, the effect of VMH leptin infusion on muscle glucose uptake is dependent on melanocortin signaling because melanocortin antagonists blocked the response to leptin (Toda et al., 2009).

In adult humans, BAT is contained along the spine and behind the muscles of the lower neck and collarbone. BAT is activated by various stimuli, including exposure to cold, and stimulates the metabolism of fat and sugars. This process is more obvious in the young and lean than in the old and obese, and in women rather than in men (Farmer, 2009). Subsequent to the subcutaneous transplantation of embryonic BAT, type 1 diabetes in streptozotocin-treated mice (which is associated with severely impaired glucose tolerance and significant loss of adipose tissue) is improved (Gunawardana and Piston, 2012). BAT transplantation results in euglycemia, normalized glucose tolerance, reduced tissue inflammation, and the reversal of clinical diabetes markers such as polyuria, polydipsia, and polyphagia (Gunawardana and Piston, 2012). These effects are independent of insulin but correlate with the recovery of the animals' white adipose tissue (Gunawardana and Piston, 2012). In uncontrolled diabetes, icv administration of leptin activates BAT, which leads to normalize blood glucose levels in STZ-induced diabetic rats. The suggested mechanism is that central leptin increases the glucose uptake to BAT, which is accompanied by the return of reduced plasma thyroxine (T4) levels and BAT uncoupling protein-1 (Ucp1) mRNA levels (Matsen et al., 2013).

### Effects of leptin on pancreatic β-cells

LepRs are present in pancreatic β-cells, and leptin inhibits insulin biosynthesis and secretion from pancreatic β-cells. By contrast, insulin stimulates leptin secretion from adipose tissue. This hormonal regulatory feedback loop is an important adipo-insular axis. Dysfunction of this adipo-insular crosstalk plays an important role in the development of hyperinsulinemia and type 2 diabetes mellitus.

Leptin directly affects pancreatic β-cell gene expression and leads to decrease insulin secretion (Seufert et al., 1999; Seufert, 2004). Furthermore, leptin affects the β-cell mass via changes in proliferation, apoptosis, and cell growth (Marroqui et al., 2012).

Leptin suppresses the expression of preproinsulin mRNA in pancreatic β-cells. In the presence of the incretin hormone glucagon-like peptide-1 (GLP-1), which stimulates the proinsulin gene promoter, leptin inhibits the GLP-1-induced expression of preproinsulin mRNA in human islets (Seufert, 2004). Leptin suppressed GLP-1-stimulated intracellular Ca^2+^ concentrations, lead to decrease insulin secretion. In contrast, the ObR-KO islets were not response to leptin (Morioka et al., 2012).

Several signaling pathways are involved in this inhibitory role of leptin in insulin secretion. Leptin inhibits glucose transport via glucose transporter 2 (GLUT-2), thus inhibiting the subsequent events in the stimulus–secretion coupling. Leptin also activates the PI3K-dependent reorganization of the actin cytoskeleton, which opens of the K_ATP_ channels. Furthermore, leptin inhibits insulin secretion by PI3K-dependent activation of phosphodiesterase 3B (PDE3B) and by decreased cAMP levels, thus inhibiting the protein kinase A (PKA) pathway, which is regulates Ca^2+^ channels and exocytosis (Marroqui et al., 2012).

PP-1alpha, the catalytic subunit of protein phosphatase 1 (PP-1), has recently been characterized genetically as a candidate gene for type 2 diabetes. PP-1 is a key enzyme in the insulin-signaling cascade in the muscles and liver. Expression of PP-1alpha has been verified in human pancreatic sections. PP-1alpha mRNA and protein expression is down-regulated by leptin, which culminates in the reduction of PP-1 enzyme activity in INS-1 pancreatic β-cells (Kuehnen et al., 2011).

The regulation of β-cell mass, (including proliferation, neogenesis, cell size, and apoptosis), is essential for the compensatory response of the endocrine pancreas to increase insulin demand, such as occurs in obesity (Marroqui et al., 2012). The proliferative effects, as well as the antiapoptotic, and proapoptotic effects, of leptin have been observed to regulate the β-cell mass (Marroqui et al., 2012).

Thus, further studies are necessary to determine the role of leptin in the regulation of β-cell mass and glucose metabolism.

## Leptin and gene therapy

Leptin has been recognized as one of the therapeutic candidates for obesity and as a regulator of fat mass, but its therapeutic effect is limited because of the apparent development of leptin resistance and the decreased BBB transport that is, observed in obese subjects with elevated blood leptin levels. In recent studies, a single, central administration of recombinant adeno-associated virus vector encoding the leptin gene severely depletes fat and ameliorates the major symptoms of metabolic syndrome for extended periods in rodents (Kalra and Kalra, 2005).

Leptin therapy has been found to reverse hyperglycemia and to prevent mortality in several rodent models of type 1 diabetes (Chinookoswong et al., 1999; Yu et al., 2008; Fujikawa et al., 2010; Wang et al., 2010b,c; Denroche et al., 2011). Intracerebroventricular (icv) infusion of leptin reverses and greatly improves hyperglycemia, hyperglucagonemia, hyperketonemia, and polyuria caused by insulin deficiency in mice. Furthermore, icv leptin delivery improves expression of the metabolically relevant hypothalamic neuropeptides POMC, NPY, and AgRP in type 1 diabetes mice (Fujikawa et al., 2010). Leptin administration improves skeletal muscle insulin responsiveness in diet-induced insulin-resistant rats (Yaspelkis et al., 2001).

Selectively increased leptin transgene expression is induced by a single icv injection of adeno-associated viral vectors encoding leptin (rAAV-lep). An injection of recombinant adenovirus encoding leptin cDNA (Ad-lep) causes hyperleptinemia and suppresses weight gain, body fat, and blood insulin levels in normal rats and ob/ob mice (Chen et al., 1996; Muzzin et al., 1996). By contrast, recombinant methionyl human leptin (r-metHuLeptin) is a recombinant analog of human leptin that is composed of the 146 aminoacids of mature human leptin with an additional methionyl residue at the N-terminal end of the recombinant protein. The difference between the two therapeutic approaches is the continuous chronic secretion of leptin mediated by gene delivery versus the intermittent bolus delivery and rapid clearance of daily injection of rh-leptin protein (Morsy et al., 1998). The comparison of daily injection of recombinant leptin protein (rh-leptin) and adenovirus-mediated delivery of the murine or human leptin gene (Ad-leptin) for treatment of obesity in the ob/ob mouse model reveals that the rate of weight loss and the percentage satiety were significantly higher in the mice treated with Ad-leptin (Morsy et al., 1998). Adeno-associated viral vectors are particularly well suited for central leptin gene therapy because of thetr low toxicity and their ability to drive transgenice expression for extended periods (Wang et al., 2010c).

The enhanced leptin transgene expression is evoked by injection of rAAV-lep into various hypothalamic sites, including the LHA, ARC, and PVN (Koyama et al., 1998; Muzzin et al., 2000). Toda et al. demonstrated that leptin injection into the VMH increases glucose uptake into the skeletal muscle, BAT, and heart; by contrast, injection into the ARC increases glucose uptake in the BAT, with no effect on glucose uptake in the DMH or the PVH (Toda et al., 2009).

A single icv injection of rAAV-lep enhances leptin transgene expression in the hypothalamus and promptly restores euglycemia that persists along with severe insulinopenia throughout a 7-week period of observation in two monogenic diabetic models the insulin-deficient non-obese Akita mice and the hyperinsulinemic, leptin-deficient, obese ob/ob mice (Ueno et al., 2006). The enhanced leptin transgene expression depletes adipose tissue and depresses weight gain by increasing energy expenditures (White et al., 2000; Dhillon et al., 2001a,b; Bagnasco et al., 2002; Dube et al., 2002; Wang et al., 2010c). The injection of rAAV-lep increases hypothalamic leptin expression and moderates diet-induced hyperglycemia, hyperinsulinemia, and skeletal muscle insulin resistance (Buettner et al., 2000; Bagnasco et al., 2003; Lee et al., 2004).

The therapeutic effects of central leptin gene therapy have been reported in insulin-deficient diabetes and in obesity animal models, such as ob/ob, diet-induced obese mice, insulin-deficient type 1 diabetes mice, and insulin-dependent diabetes animals (Akita mice) (Dube et al., 2002; Ueno et al., 2004, 2006; Yu et al., 2008; Kalra, 2009; Kojima et al., 2009; Naito et al., 2011). LepTg:Akita mice, which are Akita mice with physiological hyperleptinemia, maintain normoglycemia, insulin hypersensitivity, improved glucose tolerance, normal levels of plasma glucagon, and urinary albumin excretion rates; furthermore, these animals exhibit improved longevity (Naito et al., 2011).

Leptin administration rapidly decreases plasma gastric ghrelin and adipocyte adiponectin. Ghrelin administration readily stimulates feeding in control animals, which is completely ineffective in rAAV-lep-treated wt mice. Thus, leptin that is locally expressed in the hypothalamus counteracts the central orexigenic effects of peripheral ghrelin (Ueno et al., 2004).

This long-term benefit on glucose homeostasis is not due to diminished energy consumption, weight and adiposity but is conferred by at least two mechanisms. First, enhanced glucose metabolism to meet the demand for the rAAV-lep-induced increased non-shivering thermogenesis mediated by BAT, and second, insulin hypersensitivity (Ueno et al., 2006).

Investigators recently attempted the intranasal administration of leptin in rats with diet-induced obesity. The intranasal administration of peptides for treating a disorders originating in the brain is not a novel concept, but only the vasopressin analog desmopressin has been used clinically by this route for several decades to treat diabetes insipidus (Kastin and Pan, 2006). Intranasal leptin induces significant weight loss and reduction of adipose tissue mass in lean and diet-induced obesity rats, which is consistent with the results of central leptin administration (Schulz et al., 2012).

Taken together, increased leptin action in the hypothalamus can induce euglycemia independent of pancreatic insulin, and central leptin reinforcement. These studies indicate that leptin is therapeutically useful for the long-term treatment of insulin-deficient diabetes and diet-induced obesity, serving as a newer adjunct therapy for treating type1 and type2 diabetes.

## Clinical trial of leptin replacement therapy

The first genetic evidence of congenital leptin deficiencies in humans with severe obesity emerged in 1997 (Montague et al., 1997). Twenty patients with congenital leptin deficiencies have been identified worldwide. Their serum leptin levels were very low despite their markedly elevated fat mass, and they presented with morbid obesity, hypertension, dyslipidemia, hyperinsulinemia, insulin resistance, and hypogonadism. Most of the patients are currently on r-metHuLeptin therapy (Vatier et al., 2012). In these subjects, r-methuLeptin reduced food intake via neural circuits that reduce the cognition of food reward and enhance the response to satiety signals (Farooqi et al., 2002, 2007; Vatier et al., 2012). After therapy with r-metHuLeptin, the adult and pediatric patients exhibited beneficial changes in their body weight, body composition, endocrine function, immunity, and neurocognitive domains (Farooqi et al., 2002; Licinio et al., 2004; Williamson et al., 2005; Paz-Filho et al., 2011). The treatment produced dramatic weight loss in all three adults, corresponding to a reduction of 44–53% of baseline weight, with a preferential loss of fat. The subjects also exhibited improvements of their disordered neuroendocrine function, including the resolution of type 2 diabetes mellitus and hypogonadism and beneficial effects on ingestive and non-ingestive behaviors (Licinio et al., 2004; Williamson et al., 2005).

Three leptin-deficient adults with a missense mutation of the leptin gene were evaluated during treatment with recombinant methionyl human leptin (r-metHuLeptin), and the therapy was found decrease insulin resistance (Paz-Filho et al., 2008a). The other study investigated the effects of leptin replacement on the macro- and micronutrient preferences in leptin-deficient adults. After leptin replacement, all the patients initially presented a marked reduction in the intake of macro- and micro-nutrients, and their mean daily caloric intake dropped to 50% with a shift toward a higher percentage consumption of fats and a decrease in the intake of carbohydrates (Licinio et al., 2007b). The other patients were studied while off and on leptin therapy at a stable body weight, and significant differences were measured in their levels of macronutrients, vitamins, and minerals (Licinio et al., 2007a).

The mechanism by which the rewarding properties of food interact with physiological satiety signals and promote overeating has not been fully elucidated. Two human patients with congenital leptin deficiencies were shown images of food before and after 7 days of leptin replacement therapy, and their brain responses were measured by functional magnetic resonance imaging (fMRI). Leptin was found to modulate neural activation in key striatal regions, suggesting that this hormone acts on neural circuits governing food intake to diminish the perception of food reward while enhancing the response to satiety signals that are generated during food consumption (Farooqi et al., 2007). In the other study, during the viewing of food-related stimuli, leptin replacement reduced brain activation in the regions linked to hunger (insular, parietal, and temporal cortices) while enhancing activation in the regions linked to inhibition and satiety, as measured by fMRI (Baicy et al., 2007). In addition to its role in metabolism, treatment with r-metHuLeptin causes changes in the neurocognitive domains, suggesting that leptin might play a cognitive-enhancing role in the developing CNS (Paz-Filho et al., 2008b).

The reported indications for r-metHuLeptin therapy include leptin deficiency syndrome and lipodystrophic syndrome linked to inactivating mutations in the leptin gene, hypothalamic amenorrhea, lipodystrophic syndrome (genetic, acquired, or linked to HIV infection) and highly active antiretroviral therapy-induced lipoatrophy (Lee et al., 2006; Brennan et al., 2009; Mulligan et al., 2009).

There are few clinical reports on the effect of leptin therapy on diabetes. Two patients with type1 diabetes with hypoleptinemia associated with acquired lipodystrophy were administered r-metHuLeptin. After 1 year of leptin therapy, their glycemic control improved (Park et al., 2008). In the other clinical study, two lipoatrophic diabetes patients received leptin therapy. Both subjects exhibited extremely low amount of body fat and decreased leptin concentrations. After the initiation of leptin therapy, their fasting plasma glucose levels normalized, and their level of glycosylated hemoglobin were reduced at 4 months and remained below 6.5% for a full year (Ebihara et al., 2004). In obese patients with type 2 diabetes and hyperleptinemia, r-metHuLeptin administration did not alter the body weight but marginally reduced the level of HbA1c (Moon et al., 2011).

Future long-term studies are necessary to evaluate the efficacy of leptin therapy in type 1 and type2 diabetes.

## Perspective of leptin therapy: leptin and cognitive function

Recent studies have demonstrated that obesity is associated with an increased risk of depression (Roberts et al., 2003). Leptin changes the brain structure, neuron excitability, and synaptic plasticity. Leptin replacement (r-metHuLeptin) increased the concentration of gray matter in the anterior cingulate gyrus, inferior parietal lobule, and cerebellum, and markedly reduced body weight in the three genetically leptin-deficient adults who had recessive mutations in their *ob* genes (Matochik et al., 2005). These effects of leptin are dramatically plastic, reversible, and regionally specific in the human brain morphology (London et al., 2011). In addition to its role in metabolism, leptin might affect cognition and mood in the developing CNS. The impaired leptin activity in the hippocampus is a clue to obesity-associated depression. Leptin administration induces antidepressive behavior in normal mice and increased the antidepressive effect in leptin-overexpressing transgenic mice with hyperleptinemia (Yamada et al., 2011). Also, leptin exhibit an antidepressant-like efficacy and ameliorates anxiety in ob/ob mice (Asakawa et al., 2003). By contrast, leptin-deficient ob/ob mice display more severely depressive behavior during the FST than do normal mice, and leptin administration substantially ameliorates this depressive behavior (Lu, 2007; Yamada et al., 2011).

Taking together, leptin replacement therapy could be therapeutic opportunities not only for obesity and diabetes, but also for obesity-related depression and anxiety (Johnston et al., 2011).

## Conclusion

The control of food intake, energy expenditure, and glucose metabolism is controlled by a complex regulatory network that depends on central and peripheral crosstalk, such as found in brain-adipo signaling. The interactions between adiposity and the central neuropeptidergic cascade downstream of leptin and the role of leptin in glucose metabolism have been revealed.

Leptin gene therapy improves type1 and type2 diabetes and diet-induced obesity in animal models, such as ob/ob, diet-induced obese mice, insulin-deficient type 1 diabetes mice, and insulin-dependent diabetes animals. In contrast, obese hyperleptinemic patients with or without type 2 diabetes do not respond to exogenously administered r-metHuLeptin. Further studies are necessary for the clinical use of the leptin therapy, and leptin gene therapy is expected to be an effective therapeutic option for obesity, diabetes, depression.

### Conflict of interest statement

The authors declare that the research was conducted in the absence of any commercial or financial relationships that could be construed as a potential conflict of interest.
